# Spontaneous Regression of Pediatric Osteochondroma: Clinical Case and Comprehensive Literature Review

**DOI:** 10.7759/cureus.93957

**Published:** 2025-10-06

**Authors:** Kazu Matsumoto, Daichi Ishimaru, Kazuki Sohmiya, Nobuo Terabayashi

**Affiliations:** 1 Department of Orthopedics, Gifu Seiryu Hospital, Gifu, JPN

**Keywords:** benign bone tumor, conservative management, distal femur, osteochondroma, pediatric orthopedics, spontaneous regression

## Abstract

Osteochondromas are the most common benign bone tumors in children, typically arising in the metaphyseal regions of long bones. While most lesions remain asymptomatic and stable, their natural history is not fully understood, and spontaneous regression is exceptionally rare. We present the case of a six-year-old boy with a solitary pedunculated osteochondroma of the distal femur who was managed conservatively. Initial radiographs demonstrated a stalk-like exostosis arising from the medial aspect of the distal femur without features of hereditary multiple exostoses. The patient remained clinically stable, and serial imaging revealed progressive reduction in tumor size, with marked regression noted at three years and near-complete resolution by six years. Throughout follow-up, the patient reported no pain or functional limitation.

A review of 38 published cases, including the present case, showed that regression occurs predominantly in skeletally immature patients, with a male predominance (76.3%), solitary lesions in most cases (97.4%), and the distal femur and proximal humerus as the most frequent sites. The mean regression period was 4.2 years. Proposed mechanisms include physeal remodeling, fracture-induced remodeling, and vascular compromise, with physeal remodeling most consistent with the present case. Recognition of this rare phenomenon is important because observation with regular follow-up may be a safe alternative to surgery in asymptomatic children, thereby avoiding operative risks.

## Introduction

Osteochondromas are the most common benign bone tumors, accounting for up to 35% of all benign bone lesions [1,2]. They are classified as sessile, with a broad base, or pedunculated, with a narrow stalk [1]. Despite their prevalence, the natural history of osteochondromas remains poorly understood, as most lesions are asymptomatic and spontaneous regression is exceedingly rare.

Herein, we describe a rare case of near-complete spontaneous regression of a solitary pedunculated osteochondroma of the distal femur in a child. Importantly, we complement this case with a comprehensive literature review of all previously reported cases of regressed osteochondromas, thereby providing the most updated and systematic overview of this phenomenon. Recognition of this potential outcome is crucial to avoid unnecessary surgical intervention in children.

## Case presentation

A 6-year-old boy presented with a solitary mass around his left knee, associated with mild tenderness for several months. There was no history of trauma, systemic symptoms, or significant past medical conditions, aside from birth palsy of the right upper extremity.

On examination, a firm, mildly tender, non-adherent mass was palpable over the anteromedial aspect of the distal femur. The knee had a full range of motion, with intact neurovascular function, and no additional masses were found.

**Figure 1:**
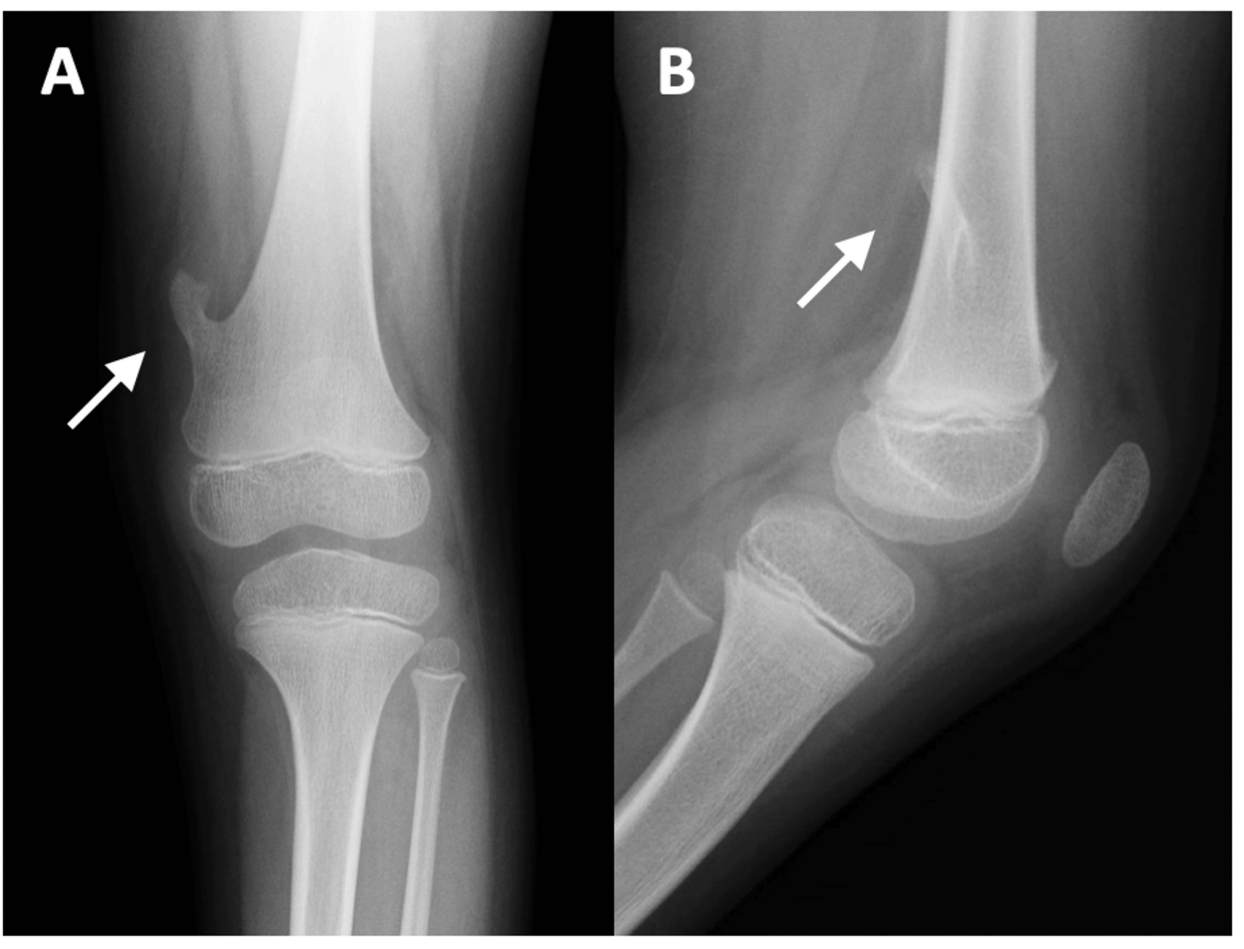
Radiographs of the osteochondroma at presentation. (A) Anteroposterior view and (B) lateral view showing a pedunculated osteochondroma with a long stalk arising from the medial aspect of the distal femur.

Plain radiographs revealed a pedunculated osteochondroma with a long stalk arising from the medial aspect of the distal femur (Figures 1A-1B). Features of hereditary multiple exostoses were absent. After multidisciplinary discussion with the family, conservative observation was chosen.

**Figure 2:**
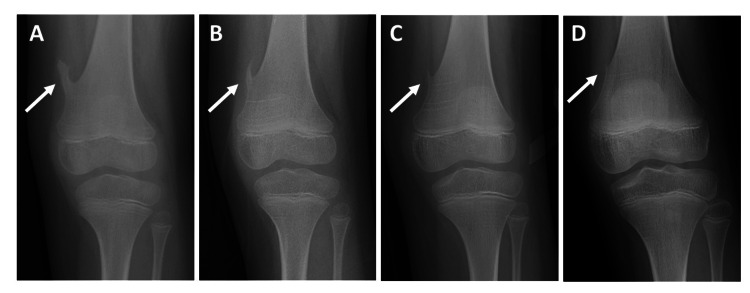
Radiographic course of spontaneous regression. (A) One-year follow-up radiograph showing a slight reduction in the osteochondroma. (B) Three-year follow-up radiograph showing a significant reduction in tumor size. (C) Four-year follow-up radiograph showing marked regression of the tumor. (D) Six-year follow-up radiograph demonstrating near-complete regression of the lesion.

At the 1-year follow-up, radiographs showed a slight reduction in the size of the osteochondroma (Figure 2A). At the 3-year follow-up, a significant reduction in tumor size was observed (Figure 2B). By 4 years, marked regression of the tumor was evident (Figure 2C). At the 6-year follow-up, near-complete regression of the lesion was demonstrated (Figure 2D), and the patient remained asymptomatic. Written informed consent for publication, including radiographs, was obtained from the patient’s parents.

## Discussion

Spontaneous regression of osteochondromas is exceptionally rare. The first case was described by Hunter J in 1835 [3]. Between 1960 and 2011, 22 cases were reported [4], and since then, an additional 16 cases, including ours, have been identified [5-32]. The current review demonstrates that regression predominantly occurs in skeletally immature patients, particularly in boys, with the distal femur and proximal humerus being the most frequent sites. This distribution corresponds to the most common anatomical locations of osteochondromas in general.

**Figure 3:**
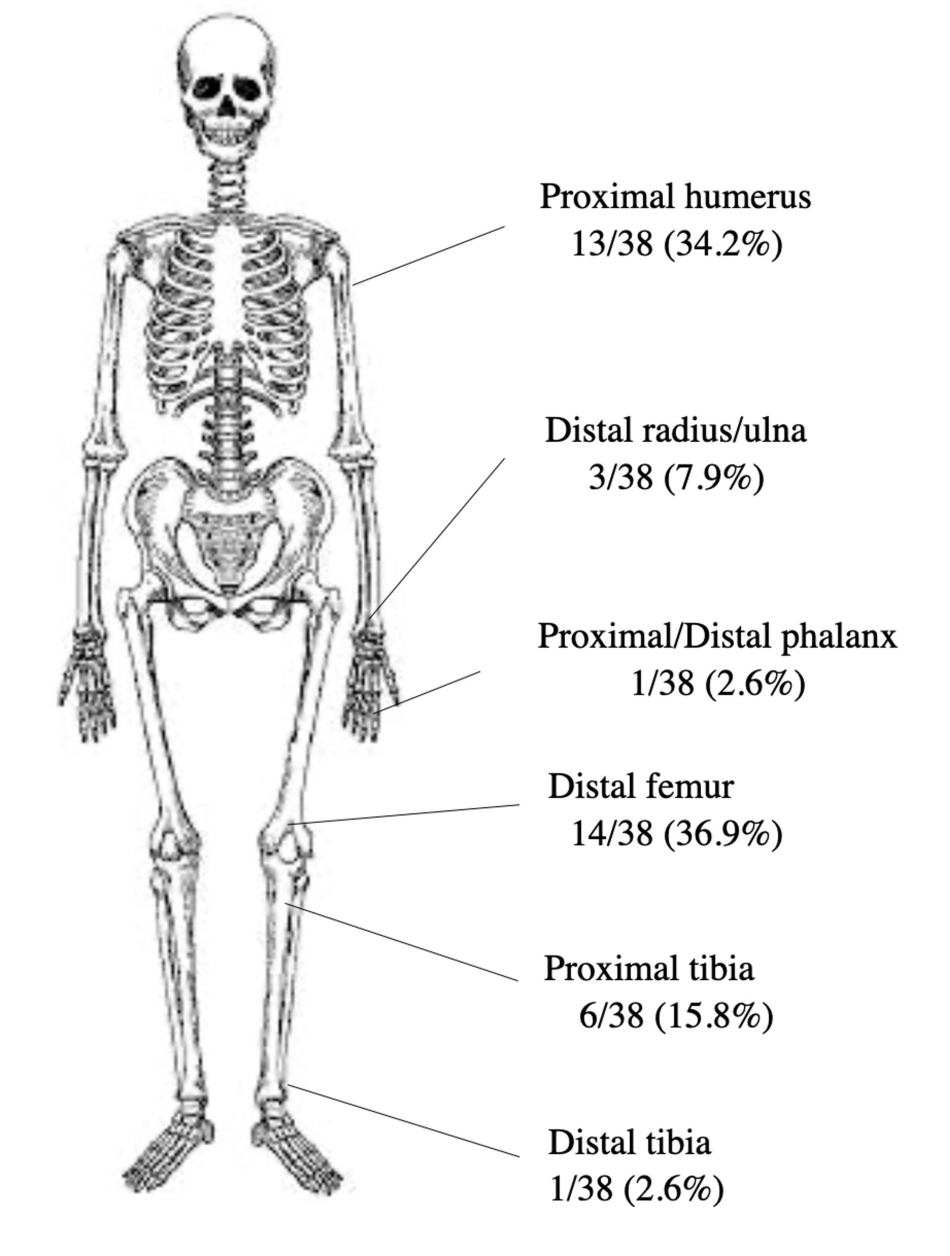
Distribution of reported regression sites. Anatomical distribution of 38 reported cases of spontaneous regression of osteochondromas, highlighting the distal femur and proximal humerus as the most commonly affected sites. This figure was created by the authors using data aggregated from published cases [5-32].

We reviewed 38 reported cases of spontaneous regression of osteochondromas, including our case (Table 1). The majority occurred in males (76.3%) and as solitary lesions (97.4%). Sessile morphology accounted for 65.8%, while 34.2% were pedunculated. The anatomical distribution is summarized in Figure 3. The distal femur (36.9%) and proximal humerus (34.2%) were the most frequent sites, followed by the proximal tibia (15.8%). Less common sites included the distal radius, distal tibia, distal ulna, and proximal phalanx. The mean age at presentation was 9.8 years, and the mean regression period was 4.2 years. A history of trauma was reported in approximately 13.2% of cases.

**Table 1:** Summary of reported cases of spontaneous regression of osteochondromas. This table summarizes 38 cases of spontaneous regression of osteochondromas reported in the literature, including the present case. The data include patient demographics, lesion morphology, anatomical location, history of trauma, and time to regression. Most cases occurred in skeletally immature male patients, predominantly presenting with solitary lesions in the distal femur and proximal humerus. The duration of regression varied widely, ranging from several months to more than a decade.

Case No.	Sex	Solitary or Multiple	Type of Osteochondroma	Location of the Lesion	Age at First Visit (yrs)	Time Until Regression (yrs)	Trauma	References
1	M	Solitary	Pedunculated	Distal femur	6	6	No	Our case
2	M	Solitary	Pedunculated	Proximal humerus	16	1.5	Yes	Moghamis IS et al. [5]
3	M	Solitary	Pedunculated	Proximal tibia	6	6	No	Adachi R et al. [6]
4	M	Solitary	Pedunculated	Distal femur	11	6	No	Le HM et al. [7]
5	M	Solitary	Sessile	Proximal humerus	11	6	No	Kalifis G Sr et al. [8]
6	M	Solitary	Sessile	Distal femur	4	3	No	Durán-Serrano M et al. [9]
7	M	Solitary	Sessile	Proximal humerus	10	3	No	-
8	M	Solitary	Pedunculated	Proximal humerus	11	4	No	-
9	M	Solitary	Sessile	Distal femur	1.3	0.5	Yes	Heyworth PB et al. [10]
10	M	Solitary	Pedunculated	Distal femur	16	3	No	Aiba H et al. [11]
11	M	Solitary	Sessile	Distal femur	7	5	No	-
12	M	Solitary	Sessile	Distal femur	6	2	No	Hill CE et al. [12]
13	M	Solitary	Sessile	Proximal humerus	6	3	No	Passanise AM et al. [13]
14	M	Solitary	Sessile	Proximal humerus	7	5	No	-
15	M	Solitary	Sessile	Proximal humerus	10	2.5	No	-
16	M	Solitary	Sessile	Distal femur	12	4	No	-
17	F	Solitary	Pedunculated	Proximal tibia	9	9	No	Mahmoodi SM et al. [14]
18	F	Solitary	Sessile	Distal femur	9	4	No	Valdivielso-Ortiz A et al. [15]
19	F	Solitary	Sessile	Distal tibia	6	2	No	Minami S et al. [16]
20	M	Multiple	Sessile	Proximal tibia	7	14	No	Yasuda H et al. [17]
21	M	Solitary	Pedunculated	Proximal humerus	7	1.25	No	Hoshi M et al. [18]
22	F	Solitary	Pedunculated	Distal femur	12	6	No	Arkader A et al. [19]
23	M	Solitary	Sessile	Distal femur	12	0.16	No	Choi JY et al. [20]
24	M	Solitary	Pedunculated	Distal femur	15	4	No	Reston SC et al. [21]
25	M	Solitary	Sessile	Distal radius	7	1.5	No	Yanagawa T et al. [22]
26	M	Solitary	Sessile	Proximal phalanx	3	6	No	Yamamoto T et al. [23]
27	F	Solitary	Sessile	Proximal humerus	9	5	No	Revilla Y et al. [24]
28	M	Solitary	Sessile	Distal ulna	7	0.5	No	Claikens B et al. [25]
29	F	Solitary	Sessile	Proximal humerus	5	2	No	Castriota-Scanderbeg A et al. [26]
30	M	Solitary	Sessile	Distal radius	12	1	Yes	-
31	M	Solitary	Pedunculated	Proximal tibia	10	3	No	Montgomery DM and LaMont RL [27]
32	M	Solitary	Sessile	Proximal humerus	11	5.5	No	-
33	M	Solitary	Sessile	Distal femur	11	2	Yes	Copeland RL et al. [28]
34	M	Solitary	Pedunculated	Distal femur	10	2.5	Yes	-
35	M	Solitary	Pedunculated	Proximal tibia	9	1	No	Paling MR [29]
36	M	Solitary	Sessile	Proximal humerus	6	6	No	Merle P et al. [30]
37	F	Solitary	Sessile	Proximal humerus	5	1	No	Callan JE et al. [31]
38	M	Solitary	Sessile	Proximal tibia	8.5	3.5	No	Sellink JL [32]

Regression was more common in sessile lesions than in pedunculated ones (60.5% vs. 39.5%), although it can occur in both morphologies. The mean regression period was approximately four years, but individual cases varied widely, ranging from a few months to over a decade, highlighting variability in biological behavior. Trauma was reported in 10.5% of cases, supporting the hypothesis that fracture-related remodeling or vascular compromise may contribute to regression in some instances.

Three mechanisms of regression have been proposed: (1) physeal remodeling as the growth plate migrates away from the lesion [25, 26], (2) remodeling following fracture or vascular compromise [6, 7, 18], and (3) resorption due to pseudoaneurysm formation [20]. Our case most likely represents physeal remodeling, as there was no evidence of fracture or vascular injury.

The clinical implications of this review are significant. Since most regressing osteochondromas were asymptomatic and resolved without intervention, conservative management with long-term follow-up is a reasonable strategy in children, particularly for lesions in common sites such as the distal femur. Recognizing the potential for regression may help avoid premature or unnecessary surgical excision, which carries inherent risks of complications.

## Conclusions

This case, together with a systematic review of 38 regressed osteochondromas, highlights that spontaneous regression, though rare, is a real and clinically significant phenomenon, particularly in children. Our report underscores the importance of long-term observation in selected asymptomatic cases and provides one of the most comprehensive updates on this topic in recent literature. Awareness of this natural course can help guide orthopedic surgeons toward more judicious treatment decisions and prevent unnecessary surgical interventions.
